# Exploring Dementia Care in an Acute Care Setting: Perspectives of Social Workers and Nurses.

**DOI:** 10.1093/geroni/igab046.3049

**Published:** 2021-12-17

**Authors:** Ruth Dunkle, Katherine Cavagnini, Joonyoung Cho, Laura Sutherland, Helen Kales, Cathleen Connell, Amanda Leggett

**Affiliations:** 1 University of Michigan, Ann Arbor, Michigan, United States; 2 Wayne State University, Ann Arbor, Michigan, United States; 3 University of California, Davis, University of California, Davis, Sacramento, California, United States; 4 University of Michigan School of Public Health, Ann Arbor, Michigan, United States; 5 The University of Michigan, Ypsilanti, Michigan, United States

## Abstract

Nurses and social workers in acute care settings have unique perspectives about providing care to persons living with dementia (PLwD) who experience behavioral and psychological symptoms of dementia (BPSD). Their distinctive roles and training have important implications for the recovery and well-being of PLwDs during hospital stays. This study utilized the "rigorous and accelerated data reduction" (RADaR) technique to compare perspectives of social workers (n=12) and nurses (n=5) in a Midwestern tertiary care facility about their caring for PLwds with BPSD. Three major themes were identified: 1) patient engagement and coordination with family and professionals, 2) treatment and medical management, and 3) barriers to care. Similarities between social workers and nurses emerged within the themes, including the importance of family involvement and providing person centered care. Differences emerged particularly within the treatment and medical management theme, as nurses utilize medications to treat BPSD and social workers were more likely to use redirection. While there is distinctive training for nurses and social workers, both identified similar barriers to providing care to PLwDs with BPSD, including time constraints, competing demands, and lack of training on BPSD management. Results demonstrate how an understanding of the critical and complementary roles that nurses and social workers play in dementia care and work together to build a care team can inform best practices to support symptom management and quality of life in PLwDs. Continuing education and training could be beneficial for both professionals to improve the quality of care for PlwDs.

